# Molecular Investigation Confirms *Myotis* Genus Bats as Common Hosts of *Polychromophilus* in Brazil

**DOI:** 10.3390/microorganisms11061531

**Published:** 2023-06-08

**Authors:** Bruno da Silva Mathias, Guilherme Augusto Minozzo, Alexander Welker Biondo, Jaciara de Oliveira Jorge Costa, Herbert Sousa Soares, Arlei Marcili, Lilian de Oliveira Guimarães, Carolina Clares dos Anjos, Andrea Pires Dos Santos, Irina Nastassja Riediger, Alan Fecchio, Marina Galvão Bueno, João Batista Pinho, Karin Kirchgatter

**Affiliations:** 1 Programa de Pós-Graduação em Medicina Tropical, Faculdade de Medicina, Universidade de São Paulo, São Paulo 05403-000, SP, Brazil; 2 Laboratório Central de Saúde Pública do Paraná, São José dos Pinhais 83060-500, PR, Brazil; 3 Departamento de Medicina Veterinária, Universidade Federal do Paraná, Curitiba 80035-050, PR, Brazil; 4 Department of Comparative Pathobiology, Purdue University, West Lafayette, IN 47907, USA; 5 Departamento de Medicina Veterinária Preventiva e Saúde Animal, Universidade de São Paulo, São Paulo 05508-270, SP, Brazil; jaciara.jorge@usp.br (J.d.O.J.C.); amarcili@usp.br (A.M.); 6 Programa de Medicina e Bem-Estar Animal e Saúde Única, Universidade Santo Amaro, São Paulo 04829-300, SP, Brazil; hesoares@prof.unisa.br; 7 Laboratório de Bioquímica e Biologia Molecular, Instituto Pasteur, São Paulo 01027-000, SP, Brazil; 8 Centro de Investigación Esquel de Montaña y Estepa Patagónica (CIEMEP), CONICET, Universidad Nacional de la Patagonia San Juan Bosco, Esquel 9200, Chubut, Argentina; 9 Laboratório de Virologia Comparada e Ambiental (LVCA), Instituto Oswaldo Cruz, Fundação Oswaldo Cruz, Fiocruz Rio de Janeiro, Rio de Janeiro 21041-250, RJ, Brazil; 10 Programa de Pós-Graduação em Ecologia e Conservação da Biodiversidade, Universidade Federal de Mato Grosso, Cuiabá 78060-900, MT, Brazil

**Keywords:** *Polychromophilus*, bats, phylogeny, *cytb*, *clpc*, asl

## Abstract

*Plasmodium* spp. and some other blood parasites belonging to the order Haemosporida are the focus of many epidemiological studies worldwide. However, haemosporidian parasites from wild animals are largely neglected in scientific research. For example, *Polychromophilus* parasites, which are exclusive to bats, are described in Europe, Asia, Africa, and Oceania, but little is known about their presence and genetic diversity in the New World. In this study, 224 samples of bats from remaining fragments of the Atlantic Forest and Pantanal biomes, as well as urbanized areas in southern and southeastern Brazil, were analyzed for the presence of haemosporidian parasites by PCR of the mitochondrial gene that encodes cytochrome b (*cytb*). The PCR fragments of the positive samples were sequenced and analyzed by the Bayesian inference method to reconstruct the phylogenetic relationships between *Polychromophilus* parasites from bats in Brazil and other countries. Sequences from Brazilian lineages of *Polychromophilus* were recovered in a clade with sequences from *Polychromophilus murinus* and close to the one *Polychromophilus* sequence obtained in Panama, the only available sequence for the American continent. This clade was restricted to bats of the family Vespertilionidae and distinct from *Polychromophilus melanipherus*, a parasite species mainly found in bats of the family Miniopteridae. The detection of *Polychromophilus* and the genetic proximity to *P. murinus* were further confirmed with the amplification of two other genes (*clpc* and *asl*). We also found a Haemosporida parasite sequence in a sample of *Noctilio albiventris* collected in the Pantanal biome, which presents phylogenetic proximity with avian *Haemoproteus* sequences. Morphological and molecular studies are still needed to conclude and describe the *Polychromophilus* species in Brazilian *Myotis* bats in more detail and to confirm *Haemoproteus* parasites in bats. Nevertheless, these molecular results in Brazilian bats confirm the importance of studying these neglected genera.

## 1. Introduction

Bats harbor a large diversity of pathogens, causing emerging infectious diseases, including bacteria and viruses [1]. They also are hosts for protozoa, such as a variety of haemosporidian parasites (Apicomplexa: Haemosporida) [2]. The parasites belonging to the Order Haemosporida (Danilewsky, 1885) are characterized by being obligatorily heteroxenous, i.e., they need more than one host to complete their evolutionary cycle. The merogony stage of a haemosporidian occurs in vertebrate hosts (reptiles, birds, and mammals), the intermediate hosts, while the sporogony stage occurs in many species of hematophagous dipterans, the definitive hosts. Haemosporidian parasites are cosmopolitan organisms widely distributed on all continents, but their diversity and phylogenetic relationships are not well established [3].

Currently, more than 500 species of haemosporidians parasitizing different groups of vertebrate hosts have been described, and new species continue to be described [4,5]. In mammals, haemosporidian infections are mainly known from primates, rodents, ungulates, and bats [6]. Bats stand out for the diversity of haemosporidian genera that can parasitize them, demonstrating a well-established parasite-host-vector relationship [3,7,8,9].

Nine genera of the order Haemosporida have been described in bats in the Old World. *Plasmodium* Marchiafava and Celli, 1885, *Polychromophilus* Dionisi 1898, *Hepatocystis* Miller 1908, *Nycteria* Garnham and Heisch, 1953, *Bioccala* [10], *Biguetiella* [11], *Dionisia* [10], all from the Plasmodiidae family Mesnil, 1903; and *Johnsprentia* [12] and *Sprattiella* [13] from the Haemoproteidae family Doflein, 1916. The two new genera, *Johnsprentia* and *Sprattiella,* were only recently described, and only morphological data is available.

*Polychromophilus* is the most widely distributed parasite genus, mainly being found in insectivorous bats of the Miniopteridae and Vespertilionidae families in temperate areas of Europe and tropical areas in Africa, Southeast Asia, and Oceania [6]. In the New World, parasitism in bats by Haemosporida species is rare, with only one species described in South America, *Polychromophilus deanei*, found in *Myotis nigricans* in Pará State, Brazil [14]. In 2014, a *Polychromophilus* sequence was recovered from *Myotis nigricans* from Panama [15], and recently, *Polychromophilus* was reported for the first time by molecular detection in Brazilian bats [16]. Here, we aimed to screen for haemosporidian parasites in bats sampled in three Brazilian biomes, extending the survey for these parasites in the Neotropics. Moreover, we amplified the caseinolytic protease C (*clpc*) gene, present in the genome of the parasite’s apicoplast, and adenylosuccinate lyase (*asl*) nuclear gene, in addition to cytochrome b (*cytb*) to confirm the close phylogenetic relationship of *Polychromophilus* parasites in Brazil to *P. murinus*.

## 2. Materials and Methods

### 2.1. Sampling

**Figure 1:**
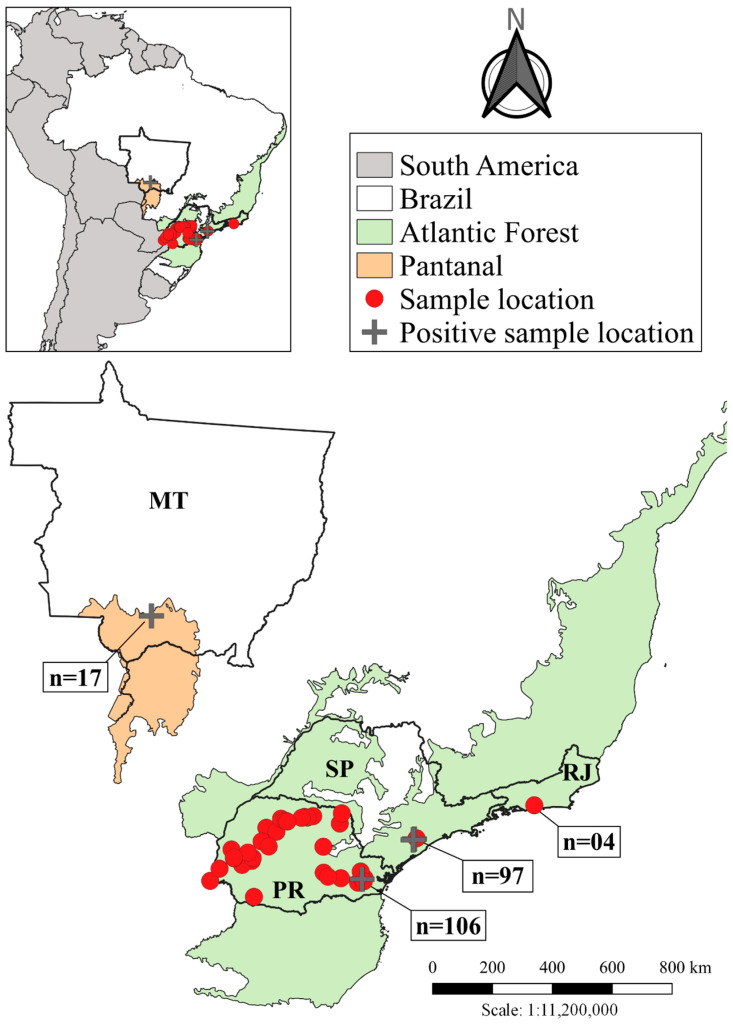
Map showing the different biomes and sample origin sites.

Samples were collected from different species of bats belonging to the Atlantic Forest and Pantanal biomes from four states of different regions, including southern (Paraná/PR), central-western (Mato Grosso/MT), and southeastern (São Paulo/SP and Rio de Janeiro/RJ) Brazil (Figure 1 and Table 1). Specific data for each state are presented in the following items.

#### 2.1.1. Paraná/PR

Brains from bats not identified to species (*n* = 96) from the Paraná State Reference Laboratory (LACEN) were acquired as part of the rabies virus circulation monitoring program. The collections were carried out between September 2019 and August 2020 in 29 different municipalities in the Paraná State, inserted in remnant fragments of the Atlantic Forest biome and urbanized areas. Samples were stored at −80 °C and thawed immediately prior to DNA/RNA extraction.

Eight blood samples from bats (six *Desmodus rotundus* and two *Diphylla ecaudata*, both family Phyllostomidae) were collected in EDTA-anticoagulated tubes by intracardiac puncture from sedated bats, between October and November 2015, in two caves in the municipality of Rio Branco do Sul, PR, which is located in a fragment of Atlantic Forest. Since they are hematophagous species, those bats were collected as part of the rabies monitoring program in herbivores (ruminants and horses) carried out by municipal and state public agencies. In addition, two non-hematophagous bats of the genus *Molossus*, family Molossidae, were collected for rabies surveillance in Curitiba city, as they were found in public areas nearby the Municipal Zoo of Curitiba, PR [17]. Blood samples were stored at −20 °C until processing.

#### 2.1.2. Mato Grosso/MT and Rio de Janeiro/RJ

Bat blood samples stored on FTA^®^ Whatman^®^ cards (Whatman, Sigma-Aldrich, Darmstadt, Germany) were collected in Mato Grosso (17 samples) and Rio de Janeiro (four samples). Blood smears were also prepared for microscopical examination from Rio de Janeiro.

In Mato Grosso, samples were collected in October 2019 in the municipality of Poconé, in rural areas within the Pantanal biome. Species and families sampled: *Glossophaga soricina*, family Phyllostomidae (five samples); *Molossus molossus*, family Molossidae (five samples); *Myotis* cf. *nigricans*, family Vespertilionidae (four samples); *Rhynchonycteris naso*, family Emballonuridae (two samples); *Noctilio albiventris*, family Noctilionidae (one sample).

Samples from Rio de Janeiro were collected in October 2020 in the city of Rio de Janeiro, in a remnant of Atlantic Forest (Pedra Branca State Park), near Fiocruz Atlantic Forest Biological Station, a highly urbanized region in the central portion of the city, under severe anthropogenic pressure. Only bats of the species *Artibeus lituratus*, family Phyllostomidae were sampled.

#### 2.1.3. São Paulo/SP

Blood samples from bats (*n* = 97) were collected in EDTA-anticoagulated tubes by intracardiac puncture from sedated bats. Certain specimens were euthanized using xylazine and ketamine, followed by inhalation of isoflurane. Subsequently, they were preserved in a 10% formaldehyde solution for future identification and storage at the Museum of Zoology of the University of São Paulo (MZUSP). From these specimens, liver (*n* = 30) and spleen (*n* = 12) were also collected.

Samples were collected from 2018 to 2021 in Legado das Águas reserve, the largest private reserve of Atlantic Forest in the country. It is inserted in the municipalities of Miracatu and Tapiraí, in the Ribeira Valley, south of the São Paulo State, located 122 km from the state capital, in the southern portion of the Serra do Mar ecological corridor. Legado das Águas covers 31,000 hectares contiguously connected to several other Conservation Units, contributing to an important ecological corridor between the coastal and inland areas of the southern region of São Paulo State. It is the largest continuous area of remaining Atlantic Forest that has suffered minimal human intervention, a factor attributed to the low demographic density and little economic development in the region.

Families and species sampled: family Phyllostomidae, *Anoura caudifer* (seven samples), *Artibeus cinereus* (two samples), *Artibeus fimbriatus* (three samples), *Artibeus gnomus* (one sample), *Artibeus lituratus* (nine samples), *Artibeus obscurus* (nine samples), *Artibeus planirostris* (three samples), *Artibeus* sp. (one sample), *Carollia perspicillata* (eighteen samples), *Chrotopterus auritus* (one sample), *Desmodus rotundus* (one sample), *Ectophylla* sp. (one sample), *Lonchorhina aurita* (one sample), *Platyrrhinus lineatus* (six samples), *Platyrrhinus* sp. (one sample), *Rhinophylla pumilio* (one sample), *Sturnira lilium* (seven samples), *Thrachops cirrhosus* (one sample), *Uroderma bilobatum* (one sample); family Emballonuridae, *Pteropteryx* sp. (three samples); family Molossidae, *Molossus ater* (one sample), *Nyctinomops* sp. (four samples); family Vespertilionidae, *Eptesicus* sp. (one sample), *Myotis nigricans* (eight samples), *Myotis riparius* (one sample), *Myotis ruber* (one sample), *Myotis* sp. (four samples). All these samples are also described in Table 1.

### 2.2. Ethics Statement

All animals and their tissue samples were collected and handled under appropriate authorizations by the Brazilian government. The project was authorized by SISBIO (Sistema de Autorização e Informação em Biodiversidade), ICMBio/MMA (Instituto Chico Mendes de Conservação da Biodiversidade/Ministério do Meio Ambiente), numbers 72790, 51714-1, and 19037-1. The study was approved by the Ethics in Use of Animals Committee, CEUA/SESA, of the Centro de Produção e Pesquisa de Imunobiológicos—CPPI/PR (approval number 01/2019 and date of approval 3 March 2020), CEUA/FIOCRUZ (approval number LM-6/18/2021 and date of approval 14 May 2018) and CEUA/SUCEN (approval number 09/2021 and date of approval 30 September 2021). Rio de Janeiro sampling was carried out under SisGen authorization A46B0E1.

### 2.3. Optical Microscopy Diagnosis

The four blood smears available for analysis acquired from bats in Rio de Janeiro were fixed with 100% methanol within 24 h of collection and stained with 10% Giemsa solution for one hour, up to 30 days after collection [3]. The smears were examined for approximately 15–20 min, viewing 100 fields at low magnification (400×) and 100 fields at high magnification (1000×) [3], using a Leica^®^ DM3000LED light microscope. The search for parasitic blood stages was carried out following previous morphological studies on haemosporidians in wild animals [3,8].

### 2.4. DNA Extraction

Brain tissue samples from Paraná State were extracted using the BioGene DNA/RNA Viral Kit (K204-4, Bioclin, Belo Horizonte, MG, Brazil), following the manufacturer’s instructions. For the blood (200 μL), the DNA was prepared according to the Illustra Kit Mini Genomic Blood Preparation Spin (GE Healthcare, Chalfont, St. Giles, UK), according to the manufacturer’s instructions.

Samples from Rio de Janeiro and Mato Grosso, stored on FTA^®^ Whatman^®^ cards (Whatman, Sigma-Aldrich, Darmstadt, Germany), were extracted using the commercial Wizard SV 96 Genomic DNA Purification System kit (PROMEGA^®^, Madison, WI, USA), according to the manufacturer’s instructions.

For samples collected in the São Paulo State, genomic DNA was extracted from blood, liver and spleen tissues using PureLink™ Genomic DNA Mini Kit (Thermo Fisher Scientific, Waltham, MA, USA), according to the manufacturer’s instructions.

### 2.5. Molecular Detection of Haemosporidian Parasites

A fragment of ~1.1 kb (approximately 92% of the gene) from the mitochondrial *cytb* gene was amplified using a nested polymerase chain reaction (PCR), taking standard precautions to prevent cross-contamination of samples. The PCR reactions were conducted as described in [18] using primers DW2 and DW4 and 5 µL (50 ng) of genomic DNA in the first reaction. An aliquot of 1 µL of the PCR product was used as a template for a nested reaction with primers DW1 and DW6.

For *clpc*, a fragment of approximately 500 bp was amplified using a nested PCR, as described in [8], using primers clpcF and clpcR and 5 µL (50 ng) of genomic DNA in the first reaction. An aliquot of 1 µL of the PCR product was used as a template for a nested reaction with primers clpcF2 and clpcR2.

For *asl*, a fragment of approximately 240 bp was amplified using a nested PCR, as described in [8], using the primers aslF and aslR and 5 µL (50 ng) of genomic DNA in the first reaction. An aliquot of 1 µL of the PCR product was used as a template for a nested reaction with primers aslF2 and aslR2.

All PCR amplifications included two controls: one positive control (DNA sample with known *Polychromophilus* sp. infection) and one negative control (ultrapure water without DNA); the last one was included to check for possible contamination and false positives. All PCR products were evaluated by running 10 μL on 1% agarose gel.

PCR products were sequenced using BigDye^®^ Terminator v3.1 Cycle Sequencing Kit in ABI PRISM^®^ 3500 Genetic Analyzer (Applied Biosystems, Carlsbad, CA, USA) using nested PCR primers. For *cytb*, the oligonucleotides DW8 and DW3 were also used for sequencing [18]. The *cytb, clpc* or *asl* sequences were obtained and aligned with the sequences available in the GenBank^®^ database.

### 2.6. Phylogenetic Analysis

The phylogenetic relationships among the haemosporidian parasites were inferred using partial sequences of *cytb* (1116 bp) or the concatenated analysis of three genes, the mitochondrial cytochrome b gene (*cytb*, 725 bp), the nuclear adenylosuccinate lyase gene (*asl*, 206 bp), and the apicoplast caseinolytic protease C gene (*clpc*, 531 bp). Sequences acquired from the GenBank^®^ database used are shown in the phylogenetic trees. The alignment was obtained using the ClustalW algorithm [19] implemented in MEGAX software [20]. The phylogenetic reconstructions were performed using the Bayesian inference method implemented in MrBayes v3.2.0 [21]. The best evolution model was selected using MEGAX software [20]. The model GTR + G + I was considered to describe the substitution pattern the best (with the lowest Bayesian Information Criterion scores). Bayesian inferences were executed with two Markov Chain Monte Carlo searches of 8 million generations, with each sampling 1 of 300 trees. After a *burn-in* of 25%, the remaining 15,002 trees were used to calculate the 50% majority-rule consensus tree. The standard deviation of split frequencies was <0.01. The phylogenies were visualized using FigTree version 1.4.0 [22].

### 2.7. Host Species Identification

A fragment with ~650 bp from the mitochondrial cytochrome c oxidase (*coi*) gene was amplified using the universal primers LCO1490 and HCO2198 [23] and PCR protocol based on [24]. Amplified fragments were sequenced directly using the corresponding flanking primers. Sequences (at least 550 bp) were entered in the BOLD platform (Barcode of Life Data system) by the option “Species Level Barcode Records”. Sequences with >99% similarity were used for species identification. For sequences showing <98% identity, the neighbor-joining (NJ) tree of K2P distances showing intra and interspecific variation generated in BOLD was analyzed. Samples were considered identified if assigned to a monophyletic group of sequences corresponding to a single species.

## 3. Results

### 3.1. Host Species Identification

We used DNA barcodes and BOLD to identify bat species in the samples obtained from LACEN. Five of the 95 tested samples did not amplify (in two independent experiments). A total of 80 (84.2%) could be identified to the species level using DNA barcoding and BOLD, and 10 (10.5%) generated ambiguous identification and were identified just to genera (one *Artibeus* sp., one *Eumops* sp., four *Molossus* sp., one *Sturnira* sp., two *Eptesicus* sp. and one *Myotis* sp.). From the samples identified to species level, 50 samples showed >99% while 30 had <99% of similarity in the BOLD database. In the last case, the BOLD NJ tree was analyzed to consider the sample identified. Ten different species were obtained: 15 specimens were Vespertilionidae (two *Myotis nigricans*, one *Myotis riparius,* and 12 *Eptesicus furinalis*); 65 bats were Molossidae (23 *Molossus rufus*, 29 *Molossus molossus*, five *Tadarida brasiliensis*, three *Promops nasutus*, three *Eumops glaucinus,* one *Molossops temminckii,* and one *Molossops neglectus*).

### 3.2. Haemosporidian Parasites and Phylogeny

The percentage of positive samples by PCR was 2.67%, with six out of 224 bats infected (Table 1). No positive samples were obtained by microscopy. Positive samples were: two brain tissue samples of *Myotis riparius* (sample IDs 198 and 607), bats belonging to the Vespertilionidae family, collected in two municipalities in the Paraná State (Curitiba and São José dos Pinhais, respectively); one spleen tissue sample of *Myotis ruber* collected in Tapiraí-SP (sample ID 125); two samples, spleen and liver tissues respectively, of *Myotis sp.* from Miracatu-SP (sample IDs 138 and 141); and a blood sample of *Noctilio albiventris* (sample ID Bat17) that belongs to the Noctilionidae family. The latter species was collected near a lake in the Pantanal of Mato Grosso, municipality of Poconé (Figure 1). Interestingly, paired samples from bats ID 125, 138 and 141 obtained variable results, with liver samples being the worst for *Polychromophilus* detection (Table 2).

**Table 1:** Bats examined in this study and the prevalence of haemosporidian infection.

Host Species	Locality	Year of Collection	Bats Examined	Bats Infected with Haemosporidians (%)
Vespertilionidae Gray, 1821				
*Myotis nigricans*	Rio Bom, Toledo-PR	2019–2020	2	0
*Myotis riparius*	São José dos Pinhais, Curitiba-PR	2019–2020	2	2 (100%)
*Eptesicus furinalis*	Assis Chateaubriand, Curitiba, Foz do Iguaçu, Maringá, Ramilândia-PR	2019–2020	12	0
*Eptesicus* sp.	Curitiba-PR	2019–2020	2	0
*Myotis* sp.	Curitiba-PR	2019–2020	1	0
*Myotis* cf. *nigricans*	Poconé-MT	2019	4	0
*Eptesicus* sp.	Miracatu-SP	2018	1	0
*Myotis nigricans*	Tapiraí, Miracatu-SP	2018–2019	8	0
*Myotis riparius*	Miracatu-SP	2019	1	0
*Myotis ruber*	Tapiraí-SP	2019	1	1 (100%)
*Myotis* sp.	Miracatu-SP	2019	4	2 (50%)
Molossidae Gervais, 1856				
*Molossus* sp.	Curitiba, Araruna-PR	2019–2020	4	0
*Molossus* sp.	Curitiba-PR	2015	2	0
*Eumops* sp.	Foz do Iguaçu-PR	2019–2020	1	0
*Molossus rufus*	Braganey, Cascavel, Céu Azul, Francisco Beltrão, Jacarezinho, Londrina, Mamborê, Maringá, Maripá, Sarandi, Telêmaco Borba, Vera Cruz do Oeste-PR	2019–2020	23	0
*Molossus molossus*	Araucária, Assis Chateaubriand, Cascavel, Curitiba, Foz do Iguaçu, Guapirama, Guaratuba, Mandaguaçu, Maringá, Paulo Frontin, Ramilândia, Sarandi-PR	2019–2020	29	0
*Molossus molossus*	Poconé-MT	2019	5	0
*Tadarida brasiliensis*	Curitiba, Imbituva, Mamborê-PR	2019–2020	5	0
*Promops nasutus*	Cascavel, União da Vitória-PR	2019–2020	3	0
*Eumops glaucinus*	Assis Chateaubriand, Foz do Iguaçu, Maringá-PR	2019–2020	3	0
*Molossops temminckii*	Foz do Iguaçu-PR	2019–2020	1	0
*Molossops neglectus*	Salto do Lontra-PR	2019–2020	1	0
*Molossus ater*	Tapiraí-SP	2018	1	0
*Nyctinomops* sp.	Tapiraí-SP	2019–2020	4	0
Phyllostomidae Gray, 1825				
*Sturnira* sp.	Curitiba-PR	2019–2020	1	0
*Artibeus* sp.	Foz do Iguaçu-PR	2019–2020	1	0
*Artibeus lituratus*	Rio de Janeiro-RJ	2020	4	0
*Desmodus rotundus*	Rio Branco do Sul-PR	2015	6	0
*Diphylla ecaudata*	Rio Branco do Sul-PR	2015	2	0
*Glossophaga soricina*	Poconé-MT	2019	5	0
*Anoura caudifer*	Tapiraí-SP	2018	7	0
*Artibeus cinereus*	Tapiraí-SP	2018	2	0
*Artibeus fimbriatus*	Tapiraí, Miracatu-SP	2018	3	0
*Artibeus gnomus*	Tapiraí-SP	2018	1	0
*Artibeus lituratus*	Tapiraí-SP	2018	9	0
*Artibeus obscurus*	Tapiraí-SP	2018	9	0
*Artibeus planirostris*	Tapiraí-SP	2018	3	0
*Artibeus* sp.	Tapiraí-SP	2018	1	0
*Carollia perspicillata*	Tapiraí, Miracatu-SP	2018	18	0
*Chrotopterus auritus*	Tapiraí-SP	2018	1	0
*Desmodus rotundus*	Tapiraí-SP	2018	1	0
*Ectophylla* sp.	Tapiraí-SP	2018	1	0
*Lonchorhina aurita*	Miracatu-SP	2018	1	0
*Platyrrhinus lineatus*	Tapiraí-SP	2020	6	0
*Platyrrhinus* sp.	Tapiraí-SP	2020	1	0
*Rhinophylla pumilio*	Tapiraí-SP	2020	1	0
*Sturnira lilium*	Tapiraí-SP	2021	7	0
*Thrachops cirrhosus*	Tapiraí-SP	2021	1	0
*Uroderma bilobatum*	Miracatu-SP	2021	1	0
Emballonuridae Gervais, 1856				
*Rhynchonycteris naso*	Poconé-MT	2019	2	0
*Pteropteryx* sp.	Tapiraí-SP	2020	3	0
Noctilionidae Gray, 1821				
*Noctilio albiventris*	Poconé-MT	2019	1	1 (100%)
unknown	Curitiba, Paulo Frontin, Rolândia, Salto do Lontra-PR	2019–2020	5	0
TOTAL			224	6 (2.67%)

PR = Paraná State; MT = Mato Grosso State; RJ = Rio de Janeiro State; SP = São Paulo State.

Sequencing of the PCR amplicon revealed that sample ID 607 (*M. riparius*) was infected with *Polychromophilus* sp. It was not possible to identify the haemosporidian parasite in sample Bat17 (*Noctilio albiventris*) since an unprecedented sequence was obtained. Its closest sequence available on GenBank^®^ (KY653763) was obtained from *Haemoproteus minutus*, infecting *Turdus merula*, a passerine collected in Lithuania [25] with a 94% identity.

**Figure 2:**
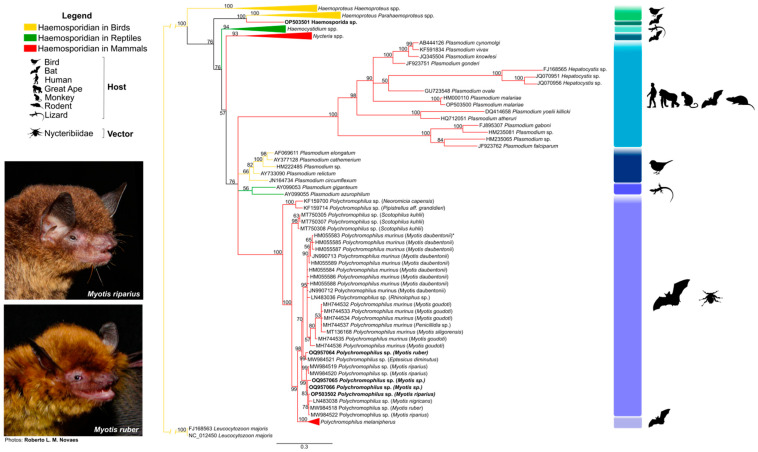
Bayesian phylogeny based on the mitochondrial cytochrome b gene (*cytb*) from haemosporidian parasites from this study and reference sequences, totaling 180 sequences (Table A1, Appendix A) in 1116 bp alignment. *Leucocytozoon* spp. was used as the external group. The support values of the nodes (in percentage) indicate posterior probabilities. The red branches highlight the haemosporidian sequences found in mammals. The yellow branches highlight the haemosporidian sequences found in birds. The green branches highlight the haemosporidian sequences found in reptiles. Sequences from this study are highlighted in bold. * Sequence HM055583 was also reported in *P. murinus* from *Eptesicus serotinus*, *Nyctalus noctula*, and *Myotis myotis*.

The *cytb* gene phylogenetic tree generated using reference sequences available in the GenBank^®^ database covering different haemosporidian genera from different hosts, as well as the sequences found herein are shown in Appendix A. *Cytb*-based phylogenetic analysis produced no conflict in any of the major nodes. All major genera and subgenera were recovered and represented in the phylogenetic tree by separate monophyletic clades (Figure 2). The results show eight clades within the order *Haemosporida* analyzed here. All *Polychromophilus* sequences from bats from different regions of the world were grouped into a monophyletic clade (posterior probability of 100) and consisted of six subclades (with posterior probabilities > 95), with all *Polychromophilus* found in Brazilian *Myotis* bats segregated in two of them (Figure 2).

The first *Polychromophilus* distinct subclade comprised two samples of *Pipistrellus aff. grandidieri* and *Neoromicia capensis*, both vespertilionid species from Guinea (KF159700 and KF159714). The second subclade contained the *Polychromophilus* sequences from *Scotophilus kuhlii*, a vespertilionid species from Thailand (MT750305, MT750307, and MT750308) (Figure 2). The third *Polychromophilus* subclade (posterior probability of 95) was composed of sequences of *P. murinus* from bats in Europe (Switzerland, Bulgaria), Madagascar, and Thailand. This subclade included a sequence of *Polychromophilus* sp. obtained in *Rhinolophus* sp. (Rhinolophidae) that stands out for not being part of the vespertilionids (Figure 2). The fourth subclade comprises sequences obtained in *Eptesicus diminutus* (MW984521) and *Myotis ruber* (OQ957064) from Brazil. The fifth subclade comprises the sequence of *Polychromophilus* obtained from *M. nigricans* from Panama and all other Brazilian sequences isolated from the genus *Myotis*. This subclade exclusively included *Polychromophilus* sequences from vespertilionids (including Brazilian ones). All *P. melanipherus* sequences from hosts of bats of the genus *Miniopterus* were distinctly separated into a subclade, confirming a clear separation of parasites from miniopterid and vespertilionid hosts (Figure 2).

The sequence of Bat17 (*N. albiventris*) clustered close to the subclade of *Haemoproteus* (*Parahaemoproteus*) spp., a specific genus of bird parasites, being positioned as a sister clade. Thus, although in the phylogenetic tree, the sequence obtained in bat was grouped with others from Haemoproteidae, it was not supported in a monophyletic clade.

The finding of *Polychromophilus* in Brazilian bats was confirmed with the amplification of the *clpc* gene, from the apicoplast of the parasite, in three samples (IDs 141, 198 and 607), presenting fragments of approximately 500 bp and also of the *asl* gene from the nuclear genome, in two samples (IDs 125 and 141), with 244 bp. Compared to sequences of the same target gene on GenBank^®^ for the genus *Polychromophilus*, the *clpc* sequences showed 97% similarities with the closest available sequences (LC715203 and LC715204), sequences from *P. murinus* described in *Myotis macrodactylus*, a bat collected in Japan [26].

**Figure 3:**
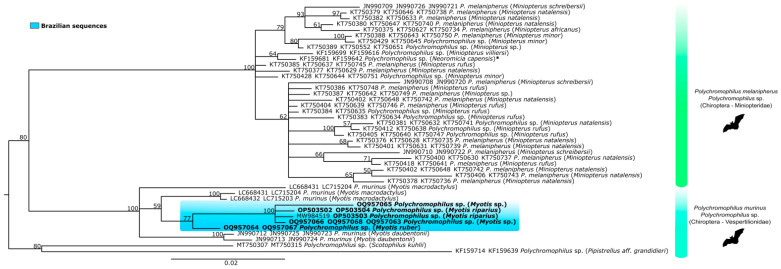
Bayesian phylogeny based on the concatenated analysis of three genes, the mitochondrial cytochrome b gene (*cytb*, 725 bp), the nuclear adenylosuccinate lyase gene (*asl*, 206 bp), and the apicoplast caseinolytic protease C gene (*clpc*, 531 bp) from *Polychromophilus* spp. of the sequences identified in the present study (highlighted in bold) and reference sequences listed in Table A2 (Appendix A), totalizing 43 sequences. The support values of the nodes (in percentage) indicate posterior probabilities. Brazilian sequences are highlighted in blue. * *Neoromicia capensis* is a vespertilionid species.

A phylogenetic tree was generated with concatenated sequences from three genes: *cytb*, *asl*, and *clpc* (Figure 3), including the *Polychromophilus* from this study and all available sequences of this genus in the GenBank^®^ database (Table A2, Appendix A). The tree topology of concatenated genes confirmed the separation of parasites from miniopterid and vespertilionid hosts, except for *Neoromicia capensis*, a vespertilionid species grouped with miniopterid hosts. The vespertilionid *Polychromophilus* subclade was divided into three branches: one with *P. murinus* sequences from Swiss bats (*Myotis daubentonii)*, one with just a sequence from *Myotis macrodactylus* from Japan, and the third with all the *Polychromophilus* found in Brazilian bats and another two from *Myotis macrodactylus* from Japan.

## 4. Discussion

The study of haemosporidian parasites in bats can significantly contribute to understanding the evolution of these parasites in mammals since seven out of nine genera of this family occurring in bats are considered specific to these hosts [6]. Haemosporidians have been found mainly in Old World bats, except for *Polychromophilus* from vespertilionid bats: *Myotis nigricans* from Brazil [14], *Myotis nigricans* from Panama [15] and, more recently, in *Myotis riparius*, *Myotis ruber* and *Eptesicus diminutus* from Brazil [16].

This study extended the search for haemosporidian parasites in bats to two additional Brazilian areas, including the Pantanal biome. We found a low haemosporidian positivity rate prevalence (2.67%), consistent with our previous study (1.2%) [16]. It is important to note that 52% of the analyzed samples were obtained from tissues (brain, spleen or liver), sample sources that are not common in haemoparasite studies but confirmed its usefulness in the screening of *Polychromophilus* parasites since we obtained the same amount of positives found in the group of blood samples.

We hypothesize that the low positivity found in our studies is related to the number of samples collected from bats of the *Myotis* genus (10% in this study), which we believe to be the main host of *Polychromophilus* in Brazil. In fact, considering only the *Myotis* bats tested, we found 21% of positives. Of all the nine samples already found positive for *Polychromophilus* by molecular methods in Brazil (this study and [16]), only one was not within the *Myotis* species.

The four new *Polychromophilus cytb* sequences obtained in this study conserved the two nucleotides T (thymine) at positions 247 and 512 of the gene, which is also observed in other Brazilian isolates, but not in the sequence from Panama [16]. Future studies analyzing the *cytb* sequence of more isolates are needed to verify whether these SNPs are molecular markers of Brazilian *Polychromophilus* isolates.

The order Chiroptera corresponds to approximately one-quarter of the mammal species in the world [27]. In Brazil, there are nine families with 182 species [28]. The Brazilian families with their respective numbers of species are Emballonuridae (17), Phyllostomidae (94), Mormoopidae (4), Noctilionidae (2), Furipteridae (1), Thyropteridae (5), Natalidae (1), Molossidae (32) and Vespetilionidae (26) [28,29]. They inhabit the entire national territory and are distributed in the most diverse biomes and urban areas, occurring in the Amazon, Cerrado, Caatinga, Atlantic Forest, Pantanal, and Pampas [28,29,30,31,32,33]. To know the diversity of bat species tested in the present study, we used DNA barcoding to identify the bat species in samples with unknown species. The results showed that most of our samples come from the Phyllostomidae family (41.5%), followed by Molossidae (36.6%), Vespertilionidae (16.9%), and Noctilionidae plus Emballonuridae (2.6%), with 2.2% unidentified. *Polychromophilus* infection in Brazilian bats continues to be limited to just one family (Vespertilionidae). However, a *Haemosporida* sp. sequence was obtained from a Noctilionidae bat (*Noctilio albiventris*), a family with just one sample analyzed. It is important to note that there is one record of *P. melanipherus* in Emballonuridae (*Taphozous melanopogon* from Thailand) but no previous record of haemosporidian parasites in Molossidae, Phyllostomidae, and Noctilionidae families. Therefore, it is very likely that the prevalence of haemosporidian parasites was low in our study because the vast majority of samples analyzed were from species that are uncommon hosts for these parasites. Since molecular studies showed that 89% of *Polychromophilus*-positive samples in Brazil were from *Myotis* species, further studies are needed to confirm their host specificity and to determine if *Myotis* spp. are the primary hosts for *Polychromophilus* in the Neotropics.

The bat *Noctilio albiventris* has a wide geographic distribution, occurring practically throughout Latin America and almost the entire Brazilian territory. It has an insectivorous diet and is always related to humid forest habitats and environments close to rivers, lakes, or coastal marine habitats [33], making this species more susceptible to parasitic diseases transmitted by vectors available in the environment. Moreover, its involvement with dipteran ectoparasites has not been shown [33], reinforcing the possibility of transmission of Haemoproteidae by ceratopogonid dipterans of the genus *Culicoides*, known vectors of *Haemoproteus* (*Parahaemoproteus*) spp. in birds, as well as *Hepatocystis* in bats [3,7].

The generalist feeding preferences of vector species could provide opportunities for cross-species transmission of *Haemoproteus* between avian and bat hosts. In this case, the *Haemosporida* sp. parasite detected in Bat17 likely represents an abortive spill-over infection [3]. In fact, detecting DNA in the blood without the demonstration of parasites in blood smears does not necessarily indicate successful infection, being plausible that its development cannot be completed in bats.

The *Haemosporida* sp. sequence described here, with the closest sequence identity of 94% with *Haemoproteus* (*Parahaemoproteus*) *minutus*, is insufficient to identify this parasite as any of those previously described in bats or other animals. However, if this finding is not a spill-over, the parasite sequence position in the phylogenetic tree points to a parasite of the Haemoproteidae family. In fact, the Haemoproteidae family harbors genera of haemosporidian parasites that are exclusive to bats, such as *Johnsprentia* and *Sprattiella*, which have not been analyzed molecularly yet, and sequences are lacking for comparison.

A combination of morphological evaluation and molecular studies are needed to conclude and further describe the *Polychromophilus* parasite lineage, as well as the *Haemosporida* sp. found in Brazilian bats. Nevertheless, these results confirm the importance of studying these neglected haemosporidian parasites in bats in Brazil.

## Figures and Tables

**Table 2:** Bats examined in this study with positive PCR results in paired samples.

Sample ID	Blood Sample	Spleen Sample	Liver Sample
125	NA	Positive	Negative
138	Positive	Positive	Negative
141	Positive	NA	Positive

NA = not available.

## Data Availability

The data presented in this study are available in Appendix A and also in the GenBank^®^ database. https://www.ncbi.nlm.nih.gov/genbank/ (accessed on 19 March 2022) (accession numbers OP503500-OP503504).

## Appendix A

**Table A1:** Mitochondrial cytochrome b (*cytb*) gene sequences used in phylogenetic analyzes and their respective GenBank^®^ accession numbers. Sequences from this study are highlighted in bold.

GenBank^®^ Accession Number	Parasite Species	Host Species	Country of Source
MN316537, MN316538	*Haemocystidium* cf. *chelodinae*	*Myuchelys georgesi*	Australia
MK976708-MK976710	*Haemocystidium pacayae*	*Podocnemis vogli*	Colombia
MH177855	*Haemocystidium ptyodactylii*	Squamate *	unknown
KT364883	*Haemocystidium* sp.	*Hemidactylus luqueorum*	Oman
KT364884	*Haemocystidium* sp.	*Ptyodactylus hasselquistii*	Oman
KX148083-KX148085	*Haemocystidium* sp.	*Kinixys erosa*	Gabon
KX148088-KX148090	*Haemocystidium* sp.	*Kinixys erosa*	Gabon
KX148086, KX148087	*Haemocystidium* sp.	*Pelusios castaneus*	Gabon
MT684458	*Haemocystidium* sp.	*Podocnemis vogli*	Colombia
MT684459	*Haemocystidium* sp.	*Trachylepis spilogaster*	Angola
MT684460	*Haemocystidium* sp.	*Rhacodactylus auriculatus*	New Caledonia
DQ630007	*Haemoproteus balmorali*	*Luscinia luscina*	Lithuania
DQ630014	*Haemoproteus balmorali*	*Muscicapa striata*	Lithuania
DQ630006	*Haemoproteus belopolskyi*	*Hippolais icterina*	Sweden
MK843310	*Haemoproteus belopolskyi*	*Hippolais icterina*	Lithuania
FJ168562	*Haemoproteus columbae*	*Columba livia*	USA
MK843311	*Haemoproteus hirundinis*	*Delichon urbicum*	Lithuania
KY653778	*Haemoproteus iwa*	*Fregata magnificens*	Ecuador
KY653760	*Haemoproteus jenniae*	*Creagrus furcatus*	Ecuador
DQ630010	*Haemoproteus lanii*	*Lanius collurio*	Russia
MK843313	*Haemoproteus lanii*	*Lanius collurio*	Lithuania
AY099045	*Haemoproteus majoris*	*Parus caeruleus*	Sweden
JN164727, JN164728	*Haemoproteus majoris*	*Sylvia atricapilla*	Spain
KU160476	*Haemoproteus minchini*	*Corythaeola cristata*	Singapore
DQ630013	*Haemoproteus minutus*	*Turdus merula*	Lithuania
KY653756	*Haemoproteus multipigmentatus*	*Zenaida galapagoensis*	Ecuador
MK843312	*Haemoproteus nucleocondensus*	*Acrocephalus arundinaceus*	Lithuania
JN164720	*Haemoproteus pallidulus*	*Sylvia atricapilla*	Spain
DQ630004, DQ630005	*Haemoproteus pallidus*	*Ficedula hypoleuca*	Sweden, Russia
JN164718, JN164719, JN164722	*Haemoproteus parabelopolskyi*	*Sylvia atricapilla*	Spain
DQ630009	*Haemoproteus payevsky*	*Acrocephalus scipaceus*	Lithuania
AY099040	*Haemoproteus sylvae*	*Acrocephalus arundinaceus*	Sweden
**OP503501**	*Haemosporida* sp.	*Noctilio albiventris* (ID Bat17)	Brazil
FJ168565	*Hepatocystis* sp.	*Pteropus hypomelanus*	USA
JQ070951, JQ070956	*Hepatocystis* sp.	*Cercopithecus nictitans*	Cameroon
FJ168563	*Leucocytozoon majoris*	*Zonotrichia leucophrys oriantha*	USA
NC_012450	*Leucocytozoon majoris*	*Zonotrichia leucophrys oriantha*	USA
KF159690	*Nycteria* sp.	*Rhinolophus landeri*	Guinea
KF159720	*Nycteria* sp.	*Rhinolophus alcyone*	Côte d’Ivoire
MK098843-MK098847	*Nycteria* sp.	*Rhinolophus sp., R. landeri*	Gabon
FJ168561	*Parahaemoproteus vireonis*	*Vireo gilvus*	USA
NC_012447	*Parahaemoproteus vireonis*	*Vireo gilvus*	USA
HQ712051	*Plasmodium atheruri*	*Atherurus africanus*	Madagascar
AY099055	*Plasmodium azurophilum*	*Anolis oculatus*	Dominica
AY377128	*Plasmodium cathemerium*	*Serinus canaria*	Germany
JN164734	*Plasmodium circumflexum*	*Sylvia atricapilla*	Spain
AB444126	*Plasmodium cynomolgi*	Monkey *	Japan
AF069611	*Plasmodium elongatum*	*Passer domesticus*	North America
JF923762	*Plasmodium falciparum*	*Cercopithecus nictitans*	Gabon
FJ895307	*Plasmodium gaboni*	*Pan* sp.	Gabon
AY099053	*Plasmodium giganteum*	*Agama agama*	Ghana
JF923751	*Plasmodium gonderi*	*Mandrillus sphinx*	Gabon
JQ345504	*Plasmodium knowlesi*	*Homo sapiens*	Malaysia
HM000110	*Plasmodium malariae*	*Pan troglodytes ellioti*	Cameroon
**OP503500**	*Plasmodium malariae*	*Homo sapiens* (ID I11)	Brazil
GU723548	*Plasmodium ovale*	*Homo sapiens*	England
AY733090	*Plasmodium relictum*	*Hemignathus virens*	USA
HM222485	*Plasmodium* sp.	*Icteria virens*	USA
HM235065	*Plasmodium* sp.	*Gorilla* sp.	Cameroon
HM235081	*Plasmodium* sp.	*Gorilla* sp.	Cameroon
KF591834	*Plasmodium vivax*	*Homo sapiens*	Congo
DQ414658	*Plasmodium yoelii killicki*	*Thamnomys rutilans*	Congo
JN990708-JN990711	*Polychromophilus melanipherus*	*Miniopterus schreibersii*	Switzerland
KJ131270-KJ131275	*Polychromophilus melanipherus*	*Miniopterus schreibersii*	Europa
KU182361-KU182367	*Polychromophilus melanipherus*	*Nycteribia schmidlii scotti*	Gabon
KU182368	*Polychromophilus melanipherus*	*Penicillidia fulvida*	Gabon
MH744504, MH744505	*Polychromophilus melanipherus*	*Miniopterus mahafaliensis*	Madagascar
MH744506, MH744519	*Polychromophilus melanipherus*	*Miniopterus griffithsi*	Madagascar
MH744508	*Polychromophilus melanipherus*	*Miniopterus griveaudi*	Madagascar
MH744522-MH744525	*Polychromophilus melanipherus*	*Miniopterus griveaudi*	Madagascar
MH744509-MH744511	*Polychromophilus melanipherus*	*Miniopterus gleni*	Madagascar
MH744518, MH744521	*Polychromophilus melanipherus*	*Miniopterus gleni*	Madagascar
MH744512, MH744526	*Polychromophilus melanipherus*	*Miniopterus manavi*	Madagascar
MH744514-MH744516	*Polychromophilus melanipherus*	*Miniopterus griveaudi*	Madagascar
MH744520	*Polychromophilus melanipherus*	*Paratriaenops furculus*	Madagascar
MH744527	*Polychromophilus melanipherus*	*Nycteribia stylidiopsis*	Madagascar
MH744528-MH744531	*Polychromophilus melanipherus*	*Penicillidia leptothrinax*	Madagascar
MK088162-MK088164, MK088168	*Polychromophilus melanipherus*	*Miniopterus orianae*	Australia
MT136167	*Polychromophilus melanipherus*	*Taphozous melanopogon*	Thailand
MW007671-MW007674	*Polychromophilus melanipherus*	*Nycteribia schmidlii scotti*	South Africa
MW007676	*Polychromophilus melanipherus*	*Nycteribia schmidlii scotti*	South Africa
MW007677	*Polychromophilus melanipherus*	*Miniopterus natalensis*	South Africa
MW007680-MW007682	*Polychromophilus melanipherus*	*Nycteribia schmidlii*	Hungary
MW007685	*Polychromophilus melanipherus*	*Nycteribia schmidlii*	Spain
MW007689	*Polychromophilus melanipherus*	*Miniopterus schreibersii*	Spain
HM055583	*Polychromophilus murinus*	*Myotis daubentonii*	Switzerland
HM055583	*Polychromophilus murinus*	*Eptesicus serotinus*	Switzerland
HM055583	*Polychromophilus murinus*	*Nyctalus noctula*	Switzerland
HM055583	*Polychromophilus murinus*	*Myotis myotis*	Switzerland
HM055584-HM055589	*Polychromophilus murinus*	*Myotis daubentonii*	Switzerland
JN990712, JN990713	*Polychromophilus murinus*	*Myotis daubentonii*	Switzerland
MH744532-MH744536	*Polychromophilus murinus*	*Myotis goudoti*	Madagascar
MH744537	*Polychromophilus murinus*	*Penicillidia* sp.	Madagascar
MT136168	*Polychromophilus murinus*	*Myotis siligorensis*	Thailand
KF159675, KF159681	*Polychromophilus* sp.	*Miniopterus villiersi*	Guinea
KF159699	*Polychromophilus* sp.	*Miniopterus villiersi*	Guinea
KF159700	*Polychromophilus* sp.	*Neoromicia capensis*	Guinea
LN483036	*Polychromophilus* sp.	*Rhinolophus* sp.	Bulgaria
LN483038	*Polychromophilus* sp.	*Myotis nigricans*	Panama
MK098848, MK098849	*Polychromophilus sp.*	*Miniopterus minor*	Gabon
**OP503502**	*Polychromophilus* sp.	*Myotis riparius* (ID 607)	Brazil
JQ995284-JQ995288	*Polychromophilus* sp.	*Miniopterus inflatus*	Gabon
KF159714	*Polychromophilus* sp.	*Pipistrellus aff. grandidieri*	Guinea
MT750305, MT750307, MT750308	*Polychromophilus* sp.	*Scotophilus kuhlii*	Thailand
MW984518	*Polychromophilus* sp.	*Myotis ruber*	Brazil
MW984519, MW984520	*Polychromophilus* sp.	*Myotis riparius*	Brazil
MW984522	*Polychromophilus* sp.	*Myotis riparius*	Brazil
MW984521	*Polychromophilus* sp.	*Eptesicus diminutus*	Brazil
**OQ957064**	*Polychromophilus* sp.	*Myotis ruber* (ID 125)	Brazil
**OQ957065**	*Polychromophilus* sp.	*Myotis* sp. (ID 138)	Brazil
**OQ957066**	*Polychromophilus* sp.	*Myotis* sp. (ID 141)	Brazil

* unreported species.

**Table A2:** Mitochondrial gene cytochrome b (*cytb*), nuclear gene adenylosuccinate lyase (*asl*) and apicoplast gene caseinolytic protease C (*clpc*) sequences from *Polychromophilus* species used in phylogenetic analyzes and their respective GenBank^®^ accession numbers. Sequences from this study are highlighted in bold.

Host Species	Parasite Species	*cytb*	*asl*	*clpc*	Country of Source
*Miniopterus schreibersii*	*Polychromophilus melanipherus*	JN990708	-	JN990720	Switzerland
*Miniopterus schreibersii*	*Polychromophilus melanipherus*	JN990709	JN990726	JN990721	Switzerland
*Miniopterus schreibersii*	*Polychromophilus melanipherus*	JN990710	-	JN990722	Switzerland
*Myotis daubentonii*	*Polychromophilus murinus*	JN990712	JN990725	JN990723	Switzerland
*Myotis daubentonii*	*Polychromophilus murinus*	JN990713	-	JN990724	Switzerland
*Miniopterus villiersi*	*Polychromophilus* sp.	KF159699	-	KF159616	Guinea
*Neoromicia capensis*	*Polychromophilus* sp.	KF159681	-	KF159642	Guinea
*Pipistrellus aff. grandidieri*	*Polychromophilus* sp.	KF159714	-	KF159639	Guinea
*Miniopterus natalensis*	*Polychromophilus melanipherus*	KT750379	KT750646	KT750738	Kenya
*Miniopterus natalensis*	*Polychromophilus melanipherus*	KT750382	KT750633	-	Kenya
*Miniopterus natalensis*	*Polychromophilus melanipherus*	KT750380	KT750647	KT750740	Kenya
*Miniopterus rufus*	*Polychromophilus melanipherus*	KT750385	KT750637	KT750745	Kenya
*Miniopterus rufus*	*Polychromophilus melanipherus*	KT750386	-	KT750748	Kenya
*Miniopterus* sp.	*Polychromophilus melanipherus*	KT750387	KT750642	KT750749	Kenya
*Miniopterus natalensis*	*Polychromophilus melanipherus*	KT750377	KT750629	-	Kenya
*Miniopterus africanus*	*Polychromophilus melanipherus*	KT750375	KT750627	KT750734	Kenya
*Miniopterus natalensis*	*Polychromophilus melanipherus*	KT750400	KT750630	KT750737	Kenya
*Miniopterus rufus*	*Polychromophilus melanipherus*	KT750403	KT750636	KT750744	Kenya
*Miniopterus rufus*	*Polychromophilus melanipherus*	KT750404	KT750639	KT750746	Kenya
*Miniopterus rufus*	*Polychromophilus melanipherus*	KT750418	KT750641	-	Kenya
*Miniopterus natalensis*	*Polychromophilus melanipherus*	KT750376	KT750628	KT750735	Kenya
*Miniopterus natalensis*	*Polychromophilus melanipherus*	KT750401	KT750631	KT750739	Kenya
*Miniopterus natalensis*	*Polychromophilus melanipherus*	KT750402	KT750648	KT750742	Kenya
*Miniopterus natalensis*	*Polychromophilus melanipherus*	KT750406	-	KT750743	Kenya
*Miniopterus natalensis*	*Polychromophilus melanipherus*	KT750378	-	KT750736	Kenya
*Miniopterus minor*	*Polychromophilus* sp.	KT750388	KT750643	KT750750	Tanzania
*Miniopterus minor*	*Polychromophilus* sp.	KT750428	KT750644	KT750751	Tanzania
*Miniopterus minor*	*Polychromophilus* sp.	KT750429	KT750645	-	Tanzania
*Miniopterus* sp.	*Polychromophilus* sp.	KT750389	KT750552	KT750651	Mozambique
*Miniopterus rufus*	*Polychromophilus* sp.	KT750384	KT750635	-	Kenya
*Miniopterus rufus*	*Polychromophilus* sp.	KT750383	KT750634	-	Kenya
*Miniopterus natalensis*	*Polychromophilus* sp.	KT750381	KT750632	KT750741	Kenya
*Miniopterus rufus*	*Polychromophilus* sp.	KT750412	KT750638	-	Kenya
*Miniopterus rufus*	*Polychromophilus* sp.	KT750405	KT750640	KT750747	Kenya
*Scotophilus kuhlii*	*Polychromophilus* sp.	MT750307	-	MT750315	Thailand
*Myotis macrodactylus*	*Polychromophilus murinus*	LC668431	-	LC715204	Japan
*Myotis macrodactylus*	*Polychromophilus murinus*	LC668432	-	LC715203	Japan
*Myotis macrodactylus*	*Polychromophilus murinus*	LC668433	-	LC715205	Japan
*Myotis riparius* (ID 198)	*Polychromophilus* sp.	MW984519	-	**OP503503**	Brazil
*Myotis riparius* (ID 607)	*Polychromophilus* sp.	**OP503502**	-	**OP503504**	Brazil
*Myotis ruber* (ID 125)	*Polychromophilus* sp.	**OQ957064**	**OQ957067**	-	Brazil
*Myotis* sp. (ID 138)	*Polychromophilus* sp.	**OQ957065**	-	-	Brazil
*Myotis* sp. (ID 141)	*Polychromophilus* sp.	**OQ957066**	**OQ957068**	**OQ957063**	Brazil
